# “Deflecting elastic prism” and unidirectional localisation for waves in chiral elastic systems

**DOI:** 10.1038/s41598-017-00054-6

**Published:** 2017-01-31

**Authors:** G. Carta, I. S. Jones, N. V. Movchan, A. B. Movchan, M. J. Nieves

**Affiliations:** 1 Liverpool John Moores University, Mechanical Engineering and Materials Research Centre, Liverpool, L3 3AF UK; 2 University of Liverpool, Department of Mathematical Sciences, Liverpool, L69 7ZL UK

## Abstract

For the first time, a design of a “deflecting elastic prism” is proposed and implemented for waves in a chiral medium. A novel model of an elastic lattice connected to a non-uniform system of gyroscopic spinners is designed to create a unidirectional wave pattern, which can be diverted by modifying the arrangement of the spinners within the medium. This important feature of the gyro-system is exploited to send a wave from a point of the lattice to any other point in the lattice plane, in such a way that the wave amplitude is not significantly reduced along the path. We envisage that the proposed model could be very useful in physical and engineering applications related to directional control of elastic waves.

## Introduction

An object is defined as “chiral” if it cannot be superimposed onto its mirror image^1^. In electromagnetism, chiral metamaterials have been designed to exhibit negative refraction^2–4^ and to achieve dynamic tunability^5^. In elasticity, chiral assemblies of structural elements have been proposed to realise an effective medium with negative Poisson’s ratio^6,7^ and to modify the dispersive properties of a lattice system through generating band-gaps and directional preference of waves^8,9^. An elastic metamaterial with a chiral microstructure was devised by Zhu *et al.* ^10^ to yield negative refraction effects at the sub-wavelength scale. More recently, Tallarico *et al.* ^11^ have designed a chiral interface by introducing tilted resonators into a triangular elastic lattice, which behaves as a flat elastic lens.

Chiral properties can be conferred on a discrete elastic medium by installing a system of gyros (or spinners), as proposed by Brun *et al.* ^12^ for the first time. Wave polarisation, dynamic anisotropy and forced response in the frequency domain for an elastic lattice with gyros were discussed extensively by Carta *et al.* ^13^. The same model was used by Wang *et al.* ^14^ to induce propagation of edge modes around defects in the transient regime. A gyroscopic metamaterial breaking time-reversal symmetry was presented by Nash *et al.* ^15^. A model with micro-inertia having effective frequency-dependent moment of inertia was developed by Milton and Willis^16^ by introducing a spinning top into a seemingly rigid body.

Dynamic properties of elastic discrete media have attracted much interest in the last two decades, since discrete systems allow for an analytical treatment. In particular, elastic lattices can be designed analytically to exhibit band-gaps at predefined frequencies^17,18^. Disorder and imperfections can also be included in the mathematical formulation of the model by using the notion of localisation factor^19–21^.

In this paper, we establish unidirectional localisation and directional control of waves in an elastic triangular lattice, where each mass is coupled with a gyroscopic spinner. For convenience, this medium will henceforth be referred to as a *gyro-system*. In a different way to previous works^12,13^, here we assume that the system of spinners consists of two types of gyros, having a periodic distribution throughout the lattice. We show that at certain frequencies and for a specific choice of the spinner constants, the gyro-system exhibits a spatially-localised unidirectional wave pattern. In other words, waves generated by a point source propagate along a single direction, keeping the rest of the medium undisturbed. For this very special feature, the gyro-system represents an example of a *parabolic metamaterial* ^22,23^. By using high-frequency homogenisation techniques^24–26^, the effective dynamic behaviour of the gyro-system can be described by parabolic partial differential equations in correspondence with the frequency for which the unidirectional wave phenomenon occurs.

**Figure 1:**
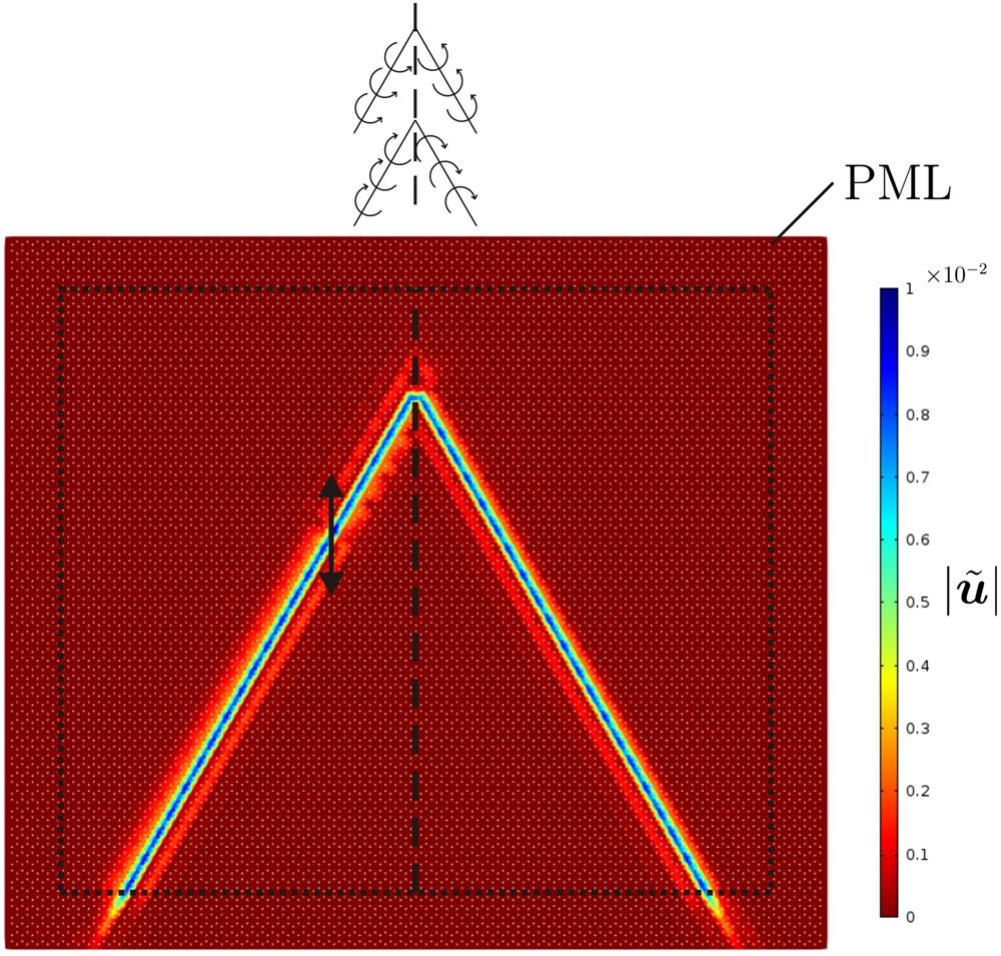
Deviation of a unidirectionally localised wave (“elastic prism”) in an elastic gyro-system. Each macro-cell contains two spinners rotating in opposite directions, as shown in Fig. 2b. The unidirectionally localised wave is generated by a harmonic displacement of amplitude 0.01, indicated by the arrow. The amplitude of the total normalised displacement, defined in the next sections, is plotted in the figure.

Figure 1 illustrates an example of unidirectional wave propagation (*elastic Gaussian beam*), which occurs when the lattice is excited by a harmonic point source at a specific frequency; this frequency can be determined from the dispersion analysis and can be varied by changing the spinning rates of the gyros. In the same model, a precise beam deviation is achieved by modifying the arrangement of the gyros within the lattice. The system is modelled as an infinite medium by attaching PML (*Perfectly Matched Layers*) to the boundaries, designed to suppress reflected waves^13,27^.

The paper is organised as follows. First, we present the model of the gyro-system and we determine its dispersion properties, with particular emphasis on the degenerate cases. Then, we show how unidirectional wave propagation can be achieved in the gyro-system. This phenomenon is subsequently exploited to send a wave from a point of the lattice to another generic point by introducing one or more interfaces in the medium. The gyro-system can be also designed to channel waves along a closed route, leading to internal resonant modes and wave amplification. Finally, we provide numerical simulations and discuss applications.

## Dispersion properties of non-uniform gyro-systems

**Figure 2:**
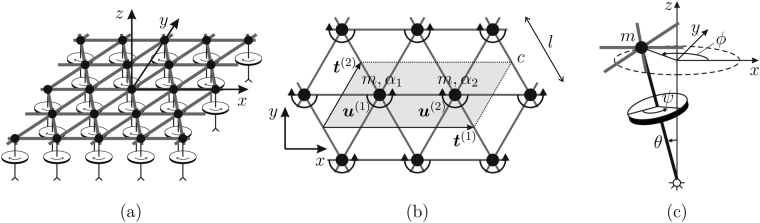
(**a**) Three-dimensional representation of the gyro-system, consisting of a triangular lattice connected to a system of gyros; (**b**) periodic unit cell of the system; (**c**) schematic representation of a gyroscopic spinner, where *ψ*, *θ* and *ϕ* are the angles of spin, nutation and precession, respectively.

We study a two-dimensional triangular lattice of masses *m*, connected by linear springs of length *l*, having stiffness *c* and negligible density. A system of gyros is attached to the lattice junctions, as shown in Fig. 2a. It consists of two types of gyros, characterised by the spinner constants *α*_1_ and *α*_2_. The unit cell of this gyro-system is sketched in Fig. 2b, where the vectors ***t*** ^(1)^ = (2*l*, 0)^T^ and ${{\boldsymbol{t}}}^{\mathrm{(2)}}={(l/\mathrm{2,}\sqrt{3l/2})}^{{\rm{T}}}$ define the periodicity of the lattice.

The axis of each gyro, which is pinned at the bottom and is free to rotate at the lattice junction, is perpendicular to the *xy*-plane in the undeformed configuration. When a lattice particle moves, the tip of the connected gyro undergoes the same displacement as the lattice particle and the spinner starts precessing (Fig. 2c). Consequently, the gyro generates a force in the plane of the lattice, perpendicular to the displacement of the lattice particle. Neglecting the forces normal to the lattice plane, such as gravity, and assuming that the displacements of the lattice particles have small amplitudes (*θ* ≪ 1), the tips of the gyros are constrained to move in the plane of the lattice^12^. A visual representation of how the lattice deforms when the lattice particles are connected to gyroscopic spinners and when they are attached to rigid rods without gyricity is provided in the Supplementary Material accompanying this paper (Video1–6). The spinner constant of a gyro, which has the dimension of mass, was evaluated by Brun *et al.* ^12^ in the time-harmonic regime by assuming that the nutation angle of the gyro has the same radian frequency *ω* as the lattice. This study is based on the equations of motion of a rigid spinner in the transient regime^28^.

### Dispersion relation

By imposing the Floquet-Bloch conditions that define the quasi-periodicity of the system, the equations of motion of the gyro-system in the time-harmonic regime can be written in compact form as^12,13^

1 $$[{\boldsymbol{C}}-{\omega }^{2}({\boldsymbol{M}}-{\boldsymbol{A}})]{\boldsymbol{U}}={\bf{0}}.$$

Here ***M*** = diag{*m*, *m*, *m*, *m*} is the mass matrix, ${\boldsymbol{U}}={({u}_{x}^{\mathrm{(1)}},{u}_{y}^{\mathrm{(1)}},{u}_{x}^{\mathrm{(2)}},{u}_{y}^{\mathrm{(2)}})}^{{\rm{T}}}$ is the displacement vector,

2 $${\boldsymbol{A}}=[\begin{matrix}0 & -\,{\rm{i}}{\alpha }_{1} & 0 & 0\\ {\rm{i}}{\alpha }_{1} & 0 & 0 & 0\\ 0 & 0 & 0 & -\,{\rm{i}}{\alpha }_{2}\\ 0 & 0 & {\rm{i}}{\alpha }_{2} & 0\end{matrix}]$$

is the spinner matrix, and ***C*** is the stiffness matrix, which depends on the wave vector ***k*** = (*k*_*x*_, *k*_*y*_)^T^. The full expression of the stiffness matrix is provided in the Supplementary Material (Eq. (S1)).

The band diagram of the system is obtained from the dispersion relation

3 $${\rm{\det }}[{\boldsymbol{C}}-{\omega }^{2}({\boldsymbol{M}}-{\boldsymbol{A}})]=0.$$

This leads to an algebraic equation of fourth order in *ω* ^2^ (Eq. (S2) in the Supplementary Material). We introduce the non-dimensional scalar quantities ${\tilde{\alpha }}_{1}={\alpha }_{1}/m$, ${\tilde{\alpha }}_{2}={\alpha }_{2}/m$, $\tilde{\omega }=\omega \sqrt{m/c}$ and the non-dimensional matrices $\tilde{{\boldsymbol{C}}}={\boldsymbol{C}}/c$ and ${\tilde{{\boldsymbol{C}}}}_{j}={{\boldsymbol{C}}}_{j}/c\,(j=1\ldots \mathrm{4)}$. The definitions of the matrices ${{\boldsymbol{C}}}_{j}\,(j=1\ldots \mathrm{4)}$ are specified in the Supplementary Material (Eqs. (S3)). Accordingly, the dispersion relation (3) can be written in non-dimensional form as

4 $$\begin{matrix} & (1-{\tilde{\alpha }}_{1}^{2})(1-{\tilde{\alpha }}_{2}^{2}){\tilde{\omega }}^{8}-(2-{\tilde{\alpha }}_{1}^{2}-{\tilde{\alpha }}_{2}^{2}){\rm{tr}}({\tilde{{\boldsymbol{C}}}}_{1}){\tilde{\omega }}^{6}\\  & \,+[(2-{\tilde{\alpha }}_{1}^{2}-{\tilde{\alpha }}_{2}^{2}){\rm{\det }}({\tilde{{\boldsymbol{C}}}}_{1})+2(1-{\tilde{\alpha }}_{1}{\tilde{\alpha }}_{2}){\rm{\det }}({\tilde{{\boldsymbol{C}}}}_{2})+{({\rm{tr}}({\tilde{{\boldsymbol{C}}}}_{1}))}^{2}\\  & \,-{({\rm{tr}}({\tilde{{\boldsymbol{C}}}}_{2}))}^{2}]{\tilde{\omega }}^{4}-2[{\rm{\det }}({\tilde{{\boldsymbol{C}}}}_{3})+{\rm{\det }}({\tilde{{\boldsymbol{C}}}}_{4})]{\tilde{\omega }}^{2}+{\rm{\det }}(\tilde{{\boldsymbol{C}}})=0.\end{matrix}$$

The components of the wave vector, appearing in the stiffness matrix, are normalised as ${\tilde{k}}_{x}={k}_{x}l$ and ${\tilde{k}}_{y}={k}_{y}l$.

### Dispersion surfaces

The solutions ${\tilde{\omega }}_{i}^{2}(i=\mathrm{1,}\ldots ,\,\mathrm{4)}$ of (4) are real for any values of ${\tilde{k}}_{x}$ and ${\tilde{k}}_{y}$, but they can be positive or negative depending on ${\tilde{\alpha }}_{1}$ and ${\tilde{\alpha }}_{2}$. We distinguish among these four cases:

- Case 1: $|{\tilde{\alpha }}_{1}|< |{\tilde{\alpha }}_{2}|< 1$. Four positive solutions ${\tilde{\omega }}_{i}(i=\mathrm{1,}\ldots ,\,\mathrm{4)}$ are admitted, which define four dispersion surfaces.
- Case 2: $|{\tilde{\alpha }}_{1}|< 1< |{\tilde{\alpha }}_{2}|$. One of the solutions for ${\tilde{\omega }}^{2}$ in (4) is negative, which implies that only three dispersion surfaces are present.
- Case 3: $1< |{\tilde{\alpha }}_{1}|< |{\tilde{\alpha }}_{2}|$. Two of the solutions for ${\tilde{\omega }}^{2}$ in (4) are negative, thus only two dispersion surfaces are defined.
- Case 4: $|{\tilde{\alpha }}_{1}|=|{\tilde{\alpha }}_{2}|=1$. This represents a degenerate case whereby (4) reduces to a quadratic equation in ${\tilde{\omega }}^{2}$, given by

5 $$[{({\rm{tr}}({\tilde{{\boldsymbol{C}}}}_{1}))}^{2}-{({\rm{tr}}({\tilde{{\boldsymbol{C}}}}_{2}))}^{2}]{\tilde{\omega }}^{4}-2[{\rm{\det }}({\tilde{{\boldsymbol{C}}}}_{3})+{\rm{\det }}({\tilde{{\boldsymbol{C}}}}_{4})]{\tilde{\omega }}^{2}+{\rm{\det }}(\tilde{{\boldsymbol{C}}})=\mathrm{0,}$$

which admits two positive solutions in $\tilde{\omega }$, yielding two dispersion surfaces.

**Figure 3:**
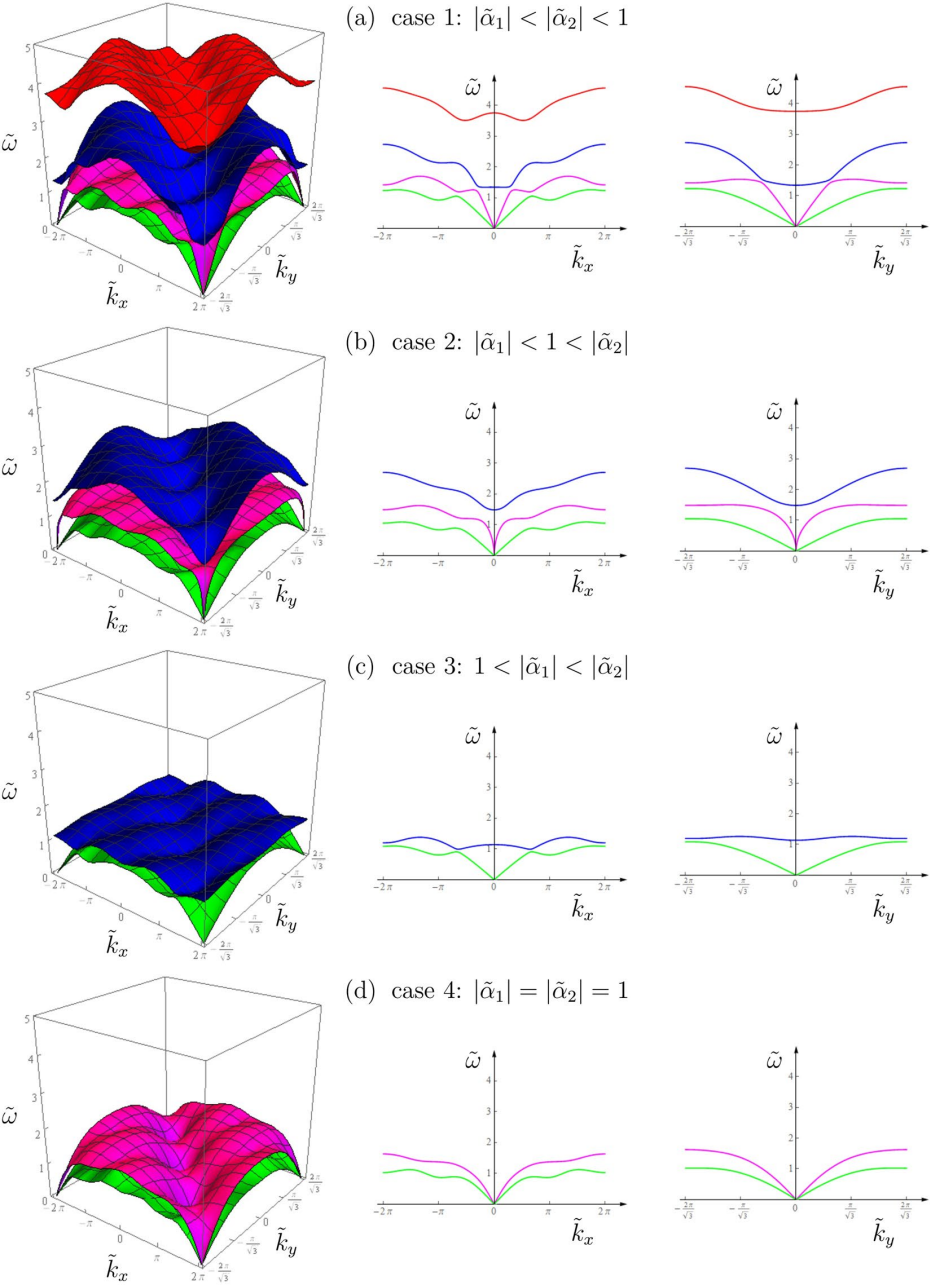
Dispersion surfaces (left) and relative cross-sections for ${\tilde{k}}_{y}=0$ (center) and ${\tilde{k}}_{x}=0$ (right) with the following values of the spinner constants: (**a**) ${\tilde{\alpha }}_{1}=0.4,{\tilde{\alpha }}_{2}=0.8$, (**b**) ${\tilde{\alpha }}_{1}=0.4,{\tilde{\alpha }}_{2}=1.6$, (**c**) ${\tilde{\alpha }}_{1}=1.2,{\tilde{\alpha }}_{2}=1.6$, (**d**) ${\tilde{\alpha }}_{1}=-\,1,{\tilde{\alpha }}_{2}=1$.

Figure 3 shows examples of the dispersion surfaces for the four cases listed above. Figure 3a illustrates the scenario when all four dispersion surfaces are present (case 1: $|{\tilde{\alpha }}_{1}|< |{\tilde{\alpha }}_{2}|< 1$). Figure 3b shows that if the absolute value of one of the spinner constants is greater than the critical value $(|{\tilde{\alpha }}_{1}|< 1< |{\tilde{\alpha }}_{2}|)$, one dispersion surface disappears. If the absolute values of both spinner constants are larger than the critical value $(|{\tilde{\alpha }}_{2}|> |{\tilde{\alpha }}_{1}|> 1)$, only two dispersion surfaces are present (Fig. 3c). When the absolute values of the spinner constants are equal to the critical value $|{\tilde{\alpha }}_{1}|=|{\tilde{\alpha }}_{2}|=1$, the dispersion relation (5) generates two dispersion surfaces (Fig. 3d).

By examining the different cases illustrated in Fig. 3, we notice that a change in the spinner constants also leads to a variation in the effective group velocities for shear and pressure waves. The latter are represented by the slopes of the lower and upper acoustic dispersion surfaces, respectively, at the origin of the dispersion diagram. The dependence of the effective group velocities on the spinner constant, together with the polarisation effect induced by the gyros, are described in depth in the paper by Carta *et al.* ^13^ for a triangular lattice connected to a uniform system of spinners. In the non-uniform gyro-system considered in this paper a new interesting phenomenon is observed, namely the possibility that the effective group velocity for pressure waves can be made infinitely large. An example is presented in Fig. 3b. This interesting feature of the non-uniform gyro-system is discussed in the Supplementary Material accompanying this paper.

## Unidirectional waveforms

In correspondence with the saddle points of the dispersion surfaces, waves in elastic lattices propagate along preferential directions. This phenomenon, known as *dynamic anisotropy*, has been observed both in lattices with and without spinners^13^,^29–33^.

The non-uniform gyro-system proposed in this paper has an even more special property: at certain frequencies, waves are channelled along one preferential direction, defined by the geometry of the lattice and by the arrangement of the gyros. In other words, the energy transferred to the system by an external point source is trapped in a line, representing a very narrow elastic Gaussian beam, while the outside field is unperturbed. This feature of the non-uniform gyro-system will be henceforth referred to with the acronym *DASER* (Dynamic Amplification by means of Spinners in an Elastic Reticulated system). In the following, we will provide some illustrative examples which show how to amplify the wave amplitude in the proposed system.

### Elastic Gaussian beam: slowness contours and forced problems

**Figure 4:**
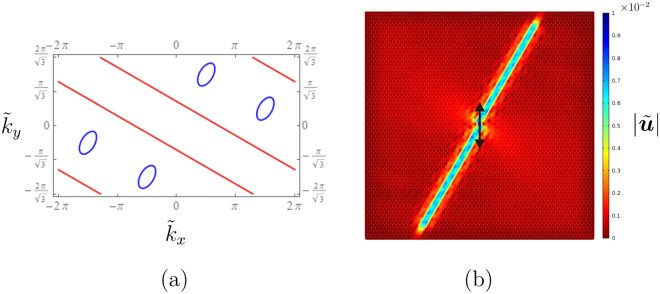
(**a**) Slowness contours and (**b**) amplitude field of the total normalised displacement, calculated at $\tilde{f}={\tilde{f}}^{{\rm{GB}}}=0.94$ in a gyro-system with equal and opposite spinner constants (${\tilde{\alpha }}_{1}=0.9,{\tilde{\alpha }}_{2}=-\,0.9$), under a vertical harmonic displacement of amplitude 0.01. The lattice in (**b**) is approximately a square of side length equal to 60 and it is surrounded by PML, the parameters of which have been tuned to maximise the reduction of reflected waves.

In a non-uniform gyro-system, it is possible to identify a frequency at which the slowness contours associated with one dispersion surface are parallel straight lines. Figure 4a shows the slowness contours determined at the non-dimensional frequency $\tilde{f}=0.94$ for a system where the two types of gyros rotate with the same spin rate but in opposite directions $({\tilde{\alpha }}_{1}=-\,{\tilde{\alpha }}_{2}=0.9)$. The slowness contours in Fig. 4a consist of parallel straight lines given by ${\tilde{k}}_{y}=-\,{\tilde{k}}_{x}/\sqrt{3}+q$ (where *q* is a constant), which represent the intersections of the plane $\tilde{f}=0.94$ with the fourth (highest) dispersion surface; the small ellipses are the intersections with the third dispersion surface. When a system is subjected to an external harmonic excitation, waves propagate along directions that are perpendicular to the slowness contours. Accordingly, at the frequency for which the slowness contours are straight lines, which will be henceforth denoted as “Gaussian beam frequency” $\tilde{f}{}^{{\rm{GB}}}$, waves propagate in the *xy*-plane of the lattice along a direction that is inclined by 60° with respect to the *x*-axis. Clearly, this direction is prescribed by the lattice geometry and by the positions of the spinners (see Fig. 2b).

Figure 4b illustrates the displacement amplitude field in a finite lattice with the same spinner constants considered above (i.e. ${\tilde{\alpha }}_{1}=-\,{\tilde{\alpha }}_{2}=0.9$), produced by a vertical harmonic displacement imposed on the central node of the system. The amplitude of the total normalised displacement, $|\tilde{{\boldsymbol{u}}}|=\sqrt{{\tilde{u}}_{x}^{2}+{\tilde{u}}_{y}^{2}}=|{\boldsymbol{u}}|/l=\sqrt{{({u}_{x}/l)}^{2}+{({u}_{y}/l)}^{2}}$, is plotted in Fig. 4b; the external excitation has amplitude $|\tilde{{\boldsymbol{u}}}|={\tilde{u}}_{y}=0.01$. The lattice is modelled as a medium of infinite extent by introducing PML (*Perfectly Matched Layers*) at the boundaries. The numerical simulations are performed by means of the finite element package *Comsol Multiphysics*.

It is apparent that the waves generated by the external source are trapped along one direction, so that the wave pattern can be described as a Gaussian beam. The same phenomenon is observed if the prescribed displacement has a direction different from the vertical one or if a harmonic force is applied instead of a harmonic displacement. The amplitude field of the normalised total displacement in logarithmic scale is presented in the Supplementary Material. There, the amplitude fields of the normalised strain energy density and kinetic energy in linear and logarithmic scales are also presented. We emphasise that the Gaussian beam phenomenon cannot be obtained in a lattice without gyros or in a gyro-system with identical spinners.

**Figure 5:**
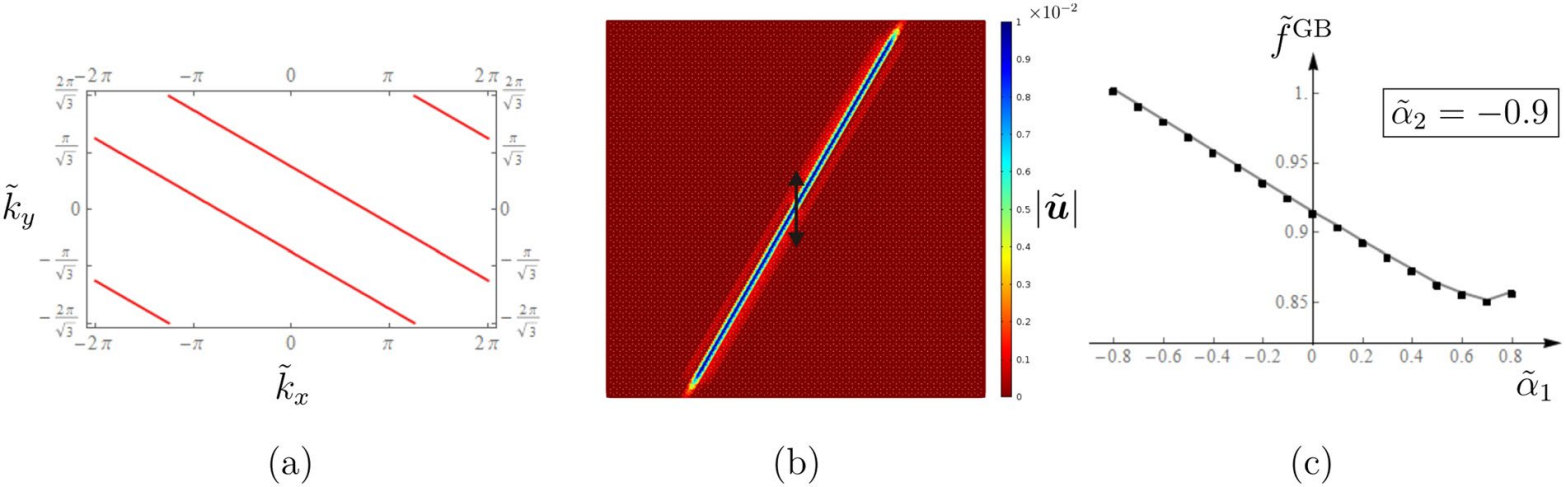
(**a**) Slowness contours and (**b**) amplitude field of the total normalised displacement, determined at $\tilde{f}={\tilde{f}}^{{\rm{GB}}}=0.86$ in a gyro-system with different spinners $({\tilde{\alpha }}_{1}=0.8,{\tilde{\alpha }}_{2}=-\,0.9)$; (**c**) dependence of the Gaussian beam frequency on the spinner constant ${\tilde{\alpha }}_{1}$ when ${\tilde{\alpha }}_{2}=-\,0.9$. The geometry of the lattice in (**b**) is the same as in Fig. 4b; the harmonic displacement has amplitude 0.01 and it is imposed on a node attached to a spinner with $\tilde{\alpha }={\tilde{\alpha }}_{2}$.

If the absolute values of the spinner constants are different and not close to each other, the Gaussian beams observed in the system are more localised. Figure 5b presents the amplitude field of the total normalised displacement in a lattice with ${\tilde{\alpha }}_{1}=0.8$ and ${\tilde{\alpha }}_{2}=-\,0.9$, computed at $\tilde{f}{}^{GB}=0.86$, when a harmonic source of amplitude $|\tilde{{\boldsymbol{u}}}|=0.01$ is applied to a lattice particle connected with a gyro having spinner constant ${\tilde{\alpha }}_{2}$. The Gaussian beam is narrower in this case because the slowness contours consist only of straight lines, since the third dispersion surface is completely below the chosen frequency (see Fig. 5a). The same conclusions are drawn by looking at the diagrams describing the distribution of the strain and kinetic energies in the medium, which are included in the Supplementary Material.

The frequency ${\tilde{f}}^{{\rm{GB}}}$ for which the slowness contours are straight lines changes with the spinner constants ${\tilde{\alpha }}_{1}$ and ${\tilde{\alpha }}_{2}$. For instance, the variation of the Gaussian beam frequency with ${\tilde{\alpha }}_{1}$ when ${\tilde{\alpha }}_{2}=-\,0.9$ is plotted in Fig. 5c. The fact that this unidirectional waveform can be produced at different frequencies by modifying the spin rates of the gyros is very important for potential practical applications.

### Double Gaussian beam

**Figure 6:**
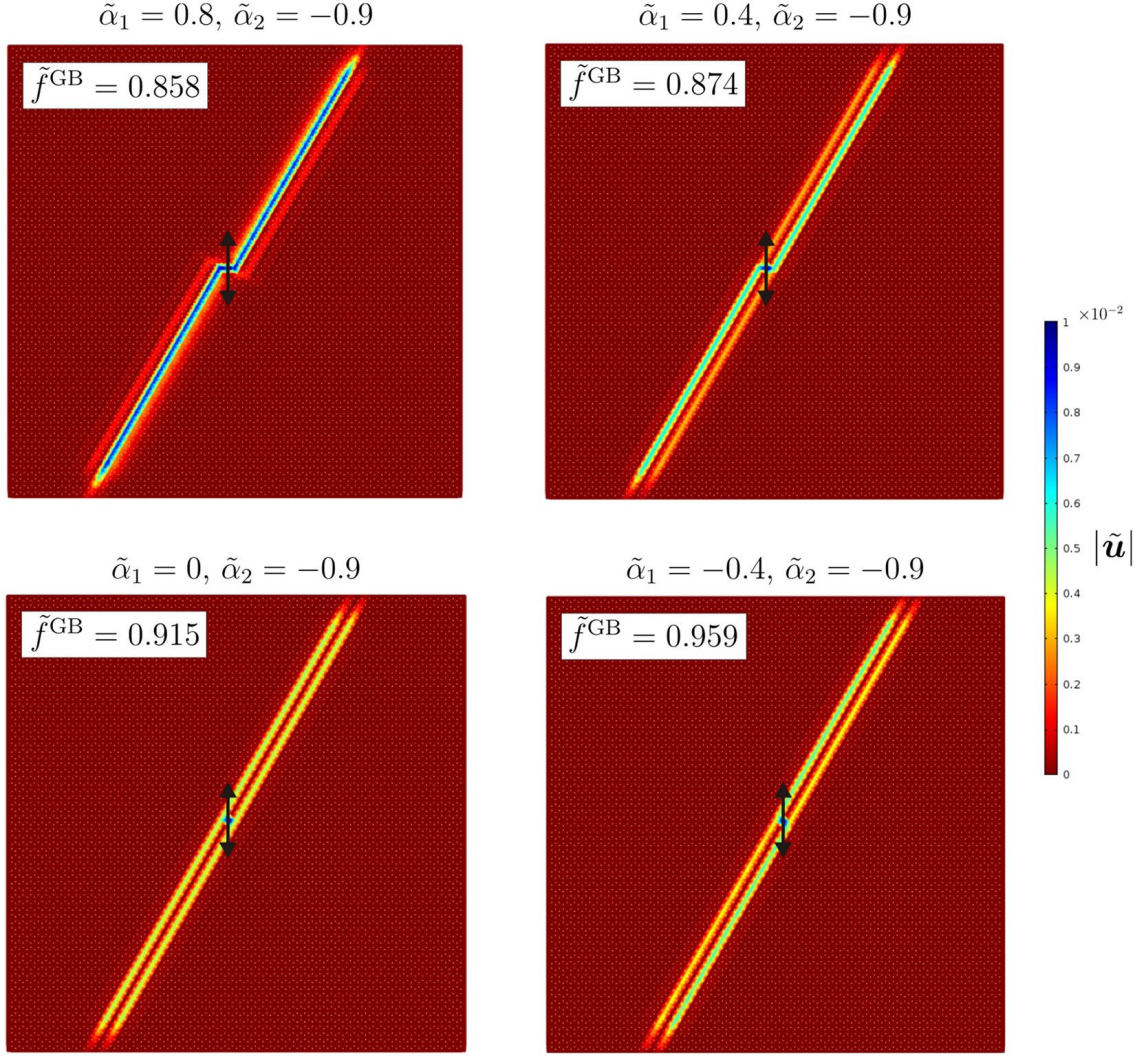
Amplitude fields of the total normalised displacement obtained for different spinner constants, resulting from a vertical harmonic displacement of amplitude 0.01 applied to a node associated with the smaller absolute value of the spinner constant.

If the external source is located at a lattice node connected with a gyro characterised by the smaller spinner constant (in absolute value), waves tend to propagate along the closest lines where the gyros with the larger absolute value of the spinner constant are placed. A demonstration is provided in Fig. 6 for different values of the spinner constants. It is interesting to notice how the intensity of the total normalised displacement amplitude redistributes from one line to the other as ${\tilde{\alpha }}_{1}$ is decreased. In particular, for ${\tilde{\alpha }}_{1}=0$ and ${\tilde{\alpha }}_{2}=-\,0.9$ we observe a double Gaussian beam, characterised by the same intensity on two parallel lines.

## Deviation of elastic Gaussian beams: “elastic prism”

Waves can be sent from one point of the lattice to any other point by appropriately arranging the gyros in the medium. This can be achieved without reducing significantly the amplitudes of the waves received at the target point.

**Figure 7:**
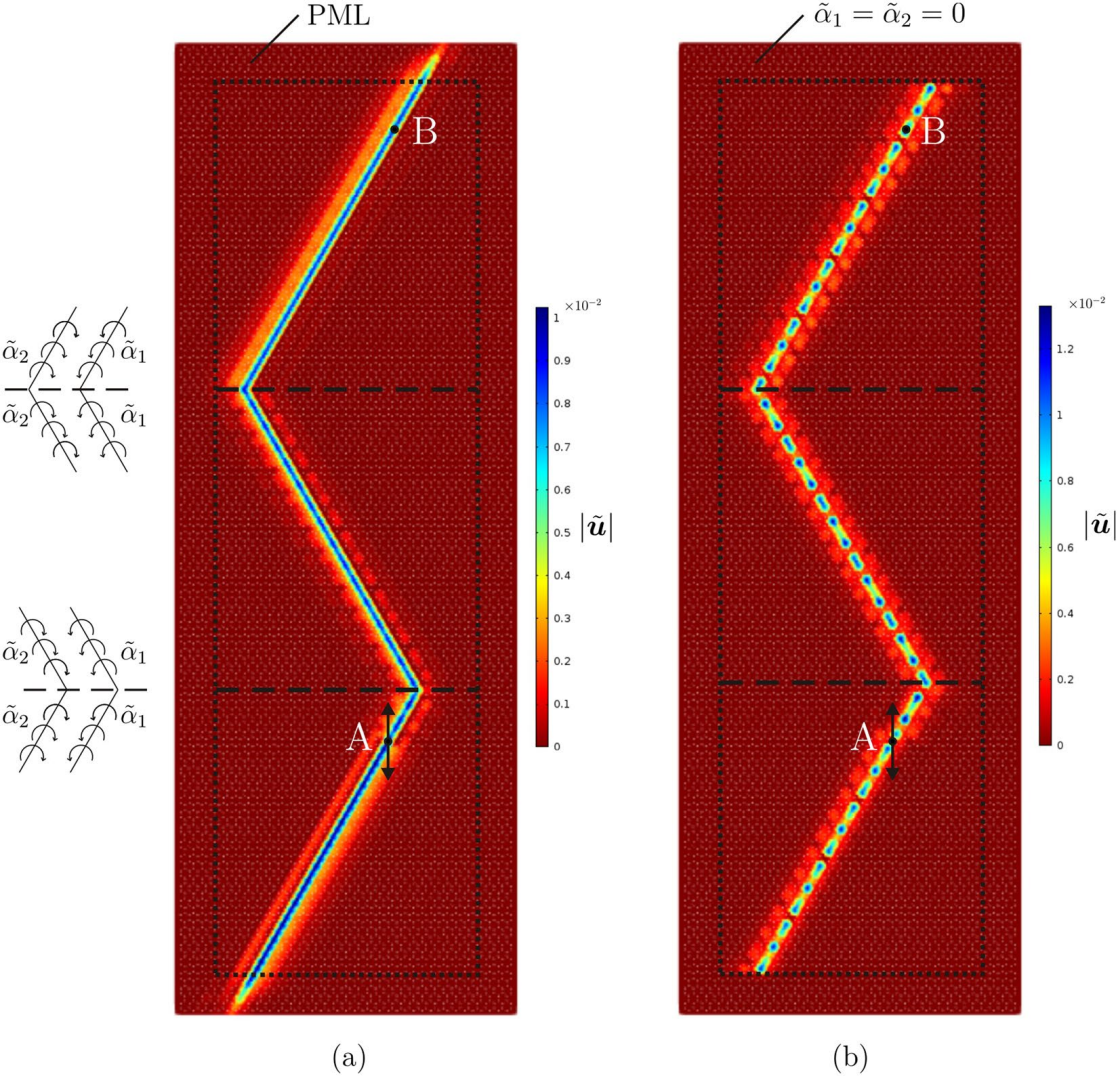
Bending of a Gaussian beam in a lattice with spinner constants ${\tilde{\alpha }}_{1}=0.8$ and ${\tilde{\alpha }}_{2}=-\,0.9$ at ${\tilde{f}}^{{\rm{GB}}}=0.858,$ when the medium is surrounded by (**a**) PML or (**b**) a lattice without gyros. The insets on the left show how the spinners are arranged in proximity of the interfaces, indicated by dashed lines.

We have previously demonstrated that, in correspondence with the Gaussian beam frequency, waves tend to propagate along the lines where the gyros with the larger absolute value of the spinner constant are located. If we want to “bend” the direction of wave propagation, we need to change the orientation of the lines with the same spinners. For instance, Fig. 7 shows that waves generated at point A can be sent to point B by introducing two interfaces, where the arrangement of the gyros is modified, as detailed in the insets of the figure.

In Fig. 7a the lattice is modelled as an infinite medium by attaching PML to the lattice sides. The maximum amplitude of the total normalised displacement is close to 0.01, which is the amplitude of the imposed displacement, and it is almost uniform along the Gaussian beam. The small discrepancies are due to the small - but not negligible - reflections occurring at the interfaces. The Gaussian beam is very localised, because the excitation is applied to a lattice point connected with a gyro characterised by the larger absolute value of the spinner constant. The same values of the parameters have been used in the model shown in Fig. 1, where a vertical interface has been introduced to deflect the Gaussian beam.

In Fig. 7b the gyro-system is surrounded by a lattice without gyros, thus it behaves as a finite system. Waves do not propagate in the lattice without gyros, because the chosen frequency (i.e. ${\tilde{f}}^{{\rm{GB}}}=0.858$) lies within its stop-band. Consequently, the waves incident on the interfaces between the two lattices are totally reflected. This explains why the maximum amplitude in the displacement field is greater than the amplitude of the imposed displacement. The difference in phase between the incident and reflected waves, due to the presence of boundaries, leads also to a modulation in the wave pattern.

The examples illustrated in Fig. 7 demonstrate that waves can be sent from one point to any other point of the lattice plane by introducing one or more interfaces, the positions of which depend on the location of the target point. This design can be applied to both a finite and an infinite medium.

## DASER: dynamic amplification

As mentioned earlier, the term DASER stands for “Dynamic Amplification by means of Spinners in an Elastic Reticulated system”.

**Figure 8:**
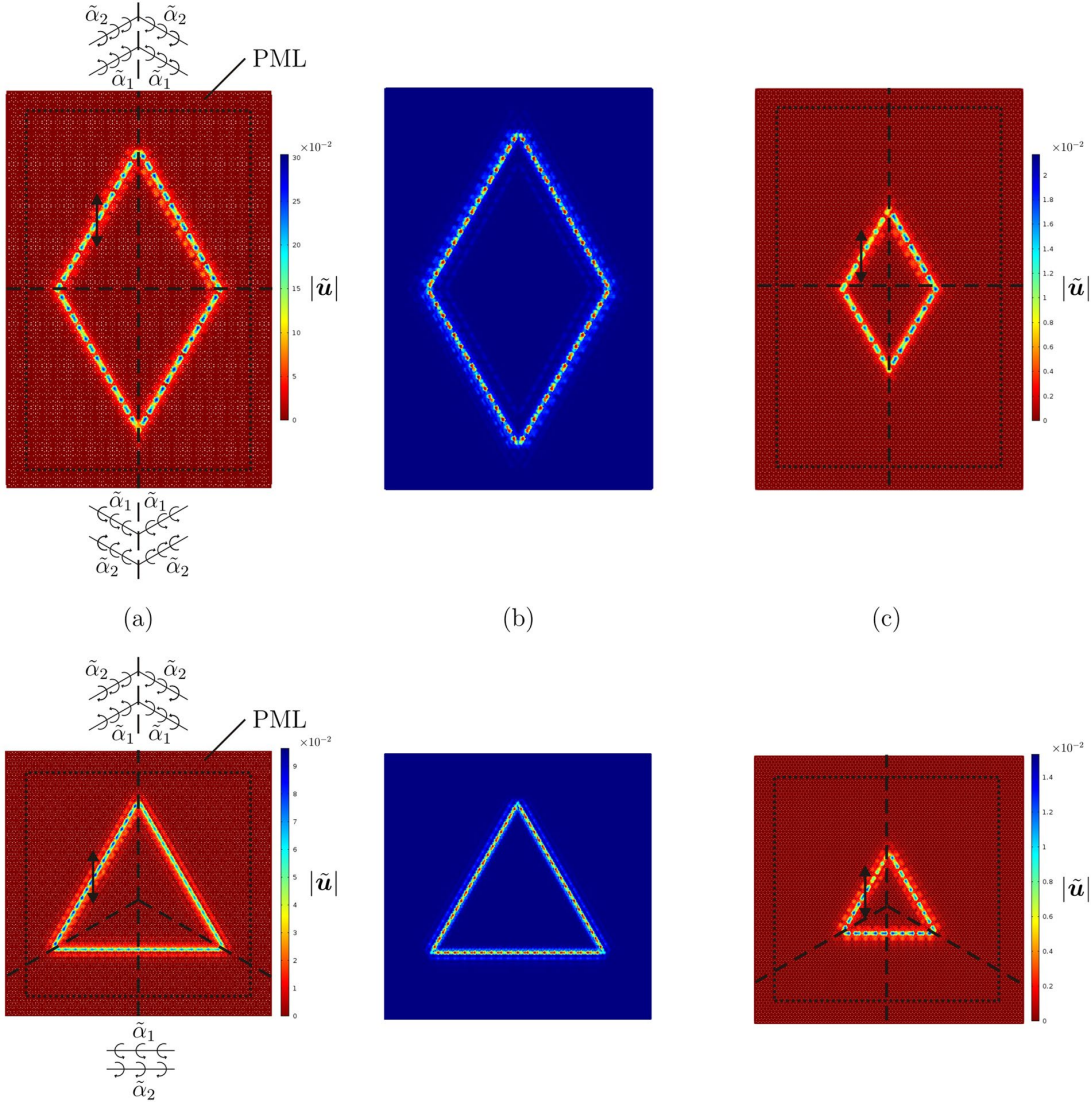
(**a**) Closed waveforms produced by a harmonic displacement of amplitude 0.01 and frequency $\,{\tilde{f}}^{{\rm{GB}}}=0.858$, indicated by the arrow, with two different configurations of the gyro-system, detailed in the figures; (**b**) resonant modes of the same lattices with Dirichlet boundary conditions in correspondence with the frequency ${\tilde{f}}^{{\rm{GB}}}$; (**c**) wave patterns obtained by placing the source at a different position.

The gyros can be positioned in the medium such that waves are forced to travel along a closed path. In Fig. 8a we have indicated how to arrange the spinners to create a rhombus and a triangle at the Gaussian beam frequency. The maximum amplitude of the waves is much larger than the amplitude of the imposed harmonic displacement due to the reflections occurring at the interfaces.

The largest wave amplitude can be obtained by placing the source in the line where resonance is expected to occur. Accordingly, in order to determine the maximum response in the lattice, we have computed the resonant modes of the system with zero displacements at the boundaries around the value of the Gaussian beam frequency, which are illustrated in Fig. 8b for both the designed configurations. By comparing Fig. 8a with Fig. 8b, we notice that the waveforms have very similar shapes. If the source is placed in a different line, the maximum amplitude of the response is smaller, as shown by an example in Fig. 8c.

The DASER phenomenon can thus be exploited to create resonant modes in an infinitely large medium and to increase considerably the amplitude of the external excitation.

## Concluding remarks

Phononic crystals and acoustic metamaterials have gained increasing attention in recent years^34–39^ due to their unique properties (e.g. sound absorption, vibration control, dynamic anisotropy, negative refraction and cloaking), which are difficult or impossible to obtain with natural materials. Similar phenomena have also been observed in systems supporting flexural waves^40–43^. Other interesting effects take place when local resonators are incorporated into a structured system^44–46^.

In this work, we have investigated the gyroscopic effects induced by a non-uniform system of spinners in an elastic lattice. While wave polarisation, band structure tunability and the disappearance of pressure waves can be attained in a system of identical spinners^12,13^, new exciting features have been discovered by allowing the system of gyros to be non-uniform. More specifically, the effective group velocity for pressure waves can be made infinitely large with an appropriate choice of the spinner constants; in addition, it is possible to produce a very localised unidirectional wave pattern. The latter phenomenon can be very useful in practical applications, as waves can be transmitted between any two points of the lattice plane without a significant reduction in the amplitude. Furthermore, waves can be channelled along closed paths, thus creating resonant effects in the medium. This important characteristic of the proposed gyro-system has been named DASER (Dynamic Amplification by means of Spinners in an Elastic Reticulated system), in analogy with the LASER in optics.

The model proposed in this paper introduces a chiral parabolic metamaterial. Examples of parabolic metamaterials in non-chiral structures were considered in recent works^22,23^. The proposed (chiral) system possesses the property of guiding elastic waves inside the medium. This phenomenon is fundamentally different from conventional waveguides, which are created by breaking periodicity and modifying the properties of the system along a prescribed path^47–51^. In contrast, in our system unidirectional propagation can be observed if we place the source at any point of the periodic lattice. In addition, the frequency at which unidirectional wave propagation occurs can be tuned by modifying the properties of the gyroscopic spinners.

## Electronic supplementary material

Supplementary Information

Supplementary Video1

Supplementary Video2

Supplementary Video3

Supplementary Video4

Supplementary Video5

Supplementary Video6
