# Supplementary material for: “Deflecting elastic prism” and unidirectional localisation for waves in chiral elastic systems
Source: Sci Rep. 2017 Jan 31;7:26. doi: 10.1038/s41598-017-00054-6 (PMC5428365; doi:10.1038/s41598-017-00054-6)
Supplement: Supplementary file 1 — Supplementary Information [file 41598_2017_54_MOESM1_ESM.pdf]

Supplementary information

Title: "Deflecting elastic prism" and unidirectional localisation for waves in chiral elastic systems

Authors: G. Carta[1,*], I.S. Jones[1], N.V. Movchan[2], A.B. Movchan[2], M.J. Nieves[1]
[1] Liverpool John Moores University, Mechanical Engineering and Materials Research Centre, Liverpool, L3 3AF, UK
[2] University of Liverpool, Department of Mathematical Sciences, Liverpool, L69 7ZL, UK
[*] Corresponding author
Email address of the corresponding author: G.Carta@ljmu.ac.uk

Videos illustrating the dynamics of the gyro-system

- Three-dimensional representation of how the periodic cell of the lattice deforms under pressure waves when the lattice points are connected to rigid rods without gyricity (Video1) and to gyroscopic spinners (Video2). The trajectories of the lattice particles are indicated in red colour.
- Two-dimensional representation of the lattice deformation under shear waves when gyros are absent (Video3) and present (Video4).
- Two-dimensional representation of the lattice deformation under pressure waves without gyros (Video5) and with gyros (Video6).

Stiffness matrix and dispersion relation

The stiffness matrix $\boldsymbol{C} = [C_{ij}]_{i,j=1}^{4}$ in (1) and (3) is given by

$$\boldsymbol{C} = c \begin{bmatrix} 3-\frac{1}{2}\cos(\zeta) & -\frac{\sqrt{3}}{2}\cos(\zeta) & -\mathrm{e}^{-\mathrm{i}(\zeta+\xi)}\begin{bmatrix} 2\cos(\zeta+\xi) \\ +\frac{1}{2}\cos(\xi)\end{bmatrix} & \frac{\sqrt{3}}{2}\mathrm{e}^{-\mathrm{i}(\zeta+\xi)}\cos(\xi) \\ -\frac{\sqrt{3}}{2}\cos(\zeta) & 3-\frac{3}{2}\cos(\zeta) & \frac{\sqrt{3}}{2}\mathrm{e}^{-\mathrm{i}(\zeta+\xi)}\cos(\xi) & -\frac{3}{2}\mathrm{e}^{-\mathrm{i}(\zeta+\xi)}\cos(\xi) \\ -\mathrm{e}^{\mathrm{i}(\zeta+\xi)}\begin{bmatrix} 2\cos(\zeta+\xi) \\ +\frac{1}{2}\cos(\xi)\end{bmatrix} & \frac{\sqrt{3}}{2}\mathrm{e}^{\mathrm{i}(\zeta+\xi)}\cos(\xi) & 3-\frac{1}{2}\cos(\zeta) & -\frac{\sqrt{3}}{2}\cos(\zeta) \\ \frac{\sqrt{3}}{2}\mathrm{e}^{\mathrm{i}(\zeta+\xi)}\cos(\xi) & -\frac{3}{2}\mathrm{e}^{\mathrm{i}(\zeta+\xi)}\cos(\xi) & -\frac{\sqrt{3}}{2}\cos(\zeta) & 3-\frac{3}{2}\cos(\zeta) \end{bmatrix}, \quad \text{(S1)}$$

where $\zeta = (k_x + k_y\sqrt{3})l/2$ and $\xi = (k_x - k_y\sqrt{3})l/2$.

The dispersion relation (3) in dimensional form is expressed as

$$\begin{aligned} &\left(m^2-\alpha_1^2\right)\left(m^2-\alpha_2^2\right)\omega^8 - m\,\mathrm{tr}\left(\boldsymbol{C}_1\right)\left(2m^2-\alpha_1^2-\alpha_2^2\right)\omega^6 \\ &+\Big\{\left(2m^2-\alpha_1^2-\alpha_2^2\right)\det\left(\boldsymbol{C}_1\right) + m^2\left[2\det\left(\boldsymbol{C}_2\right) + \left(\mathrm{tr}\left(\boldsymbol{C}_1\right)\right)^2 - \left(\mathrm{tr}\left(\boldsymbol{C}_2\right)\right)^2\right] \\ &-2\alpha_1\alpha_2\det\left(\boldsymbol{C}_2\right)\Big\}\omega^4 - 2m\left[\det\left(\boldsymbol{C}_3\right)+\det\left(\boldsymbol{C}_4\right)\right]\omega^2 + \det\left(\boldsymbol{C}\right) = 0. \end{aligned} \quad \text{(S2)}$$

The matrices $\boldsymbol{C}_j$ $(j = 1, ..., 4)$ in (S2) are defined as

$$\begin{aligned} \boldsymbol{C}_1 &= \begin{bmatrix} C_{11} & C_{12} \\ C_{12} & C_{22} \end{bmatrix} = c\begin{bmatrix} 3-\frac{1}{2}\cos(\zeta) & -\frac{\sqrt{3}}{2}\cos(\zeta) \\ -\frac{\sqrt{3}}{2}\cos(\zeta) & 3-\frac{3}{2}\cos(\zeta) \end{bmatrix}, \\ \boldsymbol{C}_2 &= \mathrm{e}^{\mathrm{i}(\zeta+\xi)}\begin{bmatrix} C_{13} & C_{14} \\ C_{14} & C_{24} \end{bmatrix} = c\begin{bmatrix} -\begin{bmatrix} 2\cos(\zeta+\xi) \\ +\frac{1}{2}\cos(\xi)\end{bmatrix} & \frac{\sqrt{3}}{2}\cos(\xi) \\ \frac{\sqrt{3}}{2}\cos(\xi) & -\frac{3}{2}\cos(\xi) \end{bmatrix}, \\ \boldsymbol{C}_3 &= \begin{bmatrix} C_{11} & C_{12} & \mathrm{e}^{\mathrm{i}(\zeta+\xi)}C_{13} \\ C_{12} & C_{22} & \mathrm{e}^{\mathrm{i}(\zeta+\xi)}C_{23} \\ \mathrm{e}^{\mathrm{i}(\zeta+\xi)}C_{13} & \mathrm{e}^{\mathrm{i}(\zeta+\xi)}C_{23} & C_{33} \end{bmatrix} = c\begin{bmatrix} 3-\frac{1}{2}\cos(\zeta) & -\frac{\sqrt{3}}{2}\cos(\zeta) & -\begin{bmatrix} 2\cos(\zeta+\xi) \\ +\frac{1}{2}\cos(\xi)\end{bmatrix} \\ -\frac{\sqrt{3}}{2}\cos(\zeta) & 3-\frac{3}{2}\cos(\zeta) & \frac{\sqrt{3}}{2}\cos(\xi) \\ -\begin{bmatrix} 2\cos(\zeta+\xi) \\ +\frac{1}{2}\cos(\xi)\end{bmatrix} & \frac{\sqrt{3}}{2}\cos(\xi) & 3-\frac{1}{2}\cos(\zeta) \end{bmatrix}, \\ \boldsymbol{C}_4 &= \begin{bmatrix} C_{22} & \mathrm{e}^{\mathrm{i}(\zeta+\xi)}C_{23} & \mathrm{e}^{\mathrm{i}(\zeta+\xi)}C_{24} \\ \mathrm{e}^{\mathrm{i}(\zeta+\xi)}C_{23} & C_{33} & C_{34} \\ \mathrm{e}^{\mathrm{i}(\zeta+\xi)}C_{24} & C_{34} & C_{44} \end{bmatrix} = c\begin{bmatrix} 3-\frac{3}{2}\cos(\zeta) & \frac{\sqrt{3}}{2}\cos(\xi) & -\frac{3}{2}\cos(\xi) \\ \frac{\sqrt{3}}{2}\cos(\xi) & 3-\frac{1}{2}\cos(\zeta) & -\frac{\sqrt{3}}{2}\cos(\zeta) \\ -\frac{3}{2}\cos(\xi) & -\frac{\sqrt{3}}{2}\cos(\zeta) & 3-\frac{3}{2}\cos(\zeta) \end{bmatrix}. \end{aligned} \quad \text{(S3)}$$

Effective group velocity for pressure waves: degenerate case

The behaviour of the gyro-system is isotropic near the origin of the dispersion diagram. In fact, at low frequencies the slowness contours are circles, given by

$$\tilde{\omega}^2 = \tilde{c}_g^2(\tilde{k}_x^2 + \tilde{k}_y^2), \tag{S4}$$

where $\tilde{c}_g = \sqrt{m/(cl^2)}\omega/|\boldsymbol{k}|$ is the non-dimensional effective group velocity.

After substituting the expression (S4) for $\tilde{\omega}$, we expand the non-dimensional dispersion relation (4) in Taylor series around the origin of the dispersion diagram up to the second order in $\tilde{k}_x$ and $\tilde{k}_y$, and we obtain the following biquadratic equation in $\tilde{c}_g$:

$$3\left[4 - (\tilde{\alpha}_1 + \tilde{\alpha}_2)^2\right]\tilde{c}_g^4 - 18\tilde{c}_g^2 + 81/16 = 0. \tag{S5}$$

In the limit when $|\tilde{\alpha}_1 + \tilde{\alpha}_2| \to 2$ one positive solution of (S5) tends to infinity: ${\tilde{c}_g}^{\mathrm{p}} \to \infty$, where ${\tilde{c}_g}^{\mathrm{p}}$ represents the effective group velocity for pressure waves. The other positive solution of (S5) ${\tilde{c}_g}^{\mathrm{s}} \to 3/(4\sqrt{2})$ when $|\tilde{\alpha}_1 + \tilde{\alpha}_2| \to 2$, where ${\tilde{c}_p}^{\mathrm{s}}$ is the effective group velocity for shear waves.

Figure S1a shows the dispersion diagram for a choice of the spinner constants $\tilde{\alpha}_1$ and $\tilde{\alpha}_2$ such that $|\tilde{\alpha}_1 + \tilde{\alpha}_2| = 2$. It is apparent that the effective group velocity for pressure waves, given by the slope of the curve in magenta at the origin, tends to infinity. The inset on the right of Fig. S1a represents the amplitude field of the total normalised displacement, $|\tilde{\boldsymbol{u}}| = \sqrt{\tilde{u}_x^2 + \tilde{u}_y^2} = |\boldsymbol{u}|/l = \sqrt{(u_x/l)^2 + (u_y/l)^2}$, generated by a harmonic displacement of amplitude 0.01, acting in the y direction, imposed on the central node of a lattice with gyros, having a low frequency $\tilde{f} = \tilde{\omega}/(2\pi) = 0.05$. The displacement amplitude field is determined by means of a finite element model developed in *Comsol Multiphysics*. PML (*Perfectly Matched Layers*) are attached to the boundaries of the lattice to minimise the amplitudes of the reflected waves, thus reproducing an infinite medium.

The wave pattern in Fig. S1a is characterised by large wavelengths, due to the high speed of pressure waves. On the other hand, Fig. S1b contains the dispersion diagram and the displacement amplitude field in a lattice where both pressure waves (the magenta curve) and shear waves (the green curve) can propagate, but where pressure waves are characterised by a lower speed with respect to the case investigated in Fig. S1a. In order to decrease the speed of pressure waves, a lower value of $\tilde{\alpha}_1$ is chosen in the second case examined. Figure S1c refers to a lattice where only shear waves can travel. For this purpose, a larger value of $\tilde{\alpha}_1$ is taken with respect to the situation in S1a. In the third case, the wavelength is much smaller in comparison with Fig. S1a, because the effects of pressure waves with high speed are absent.

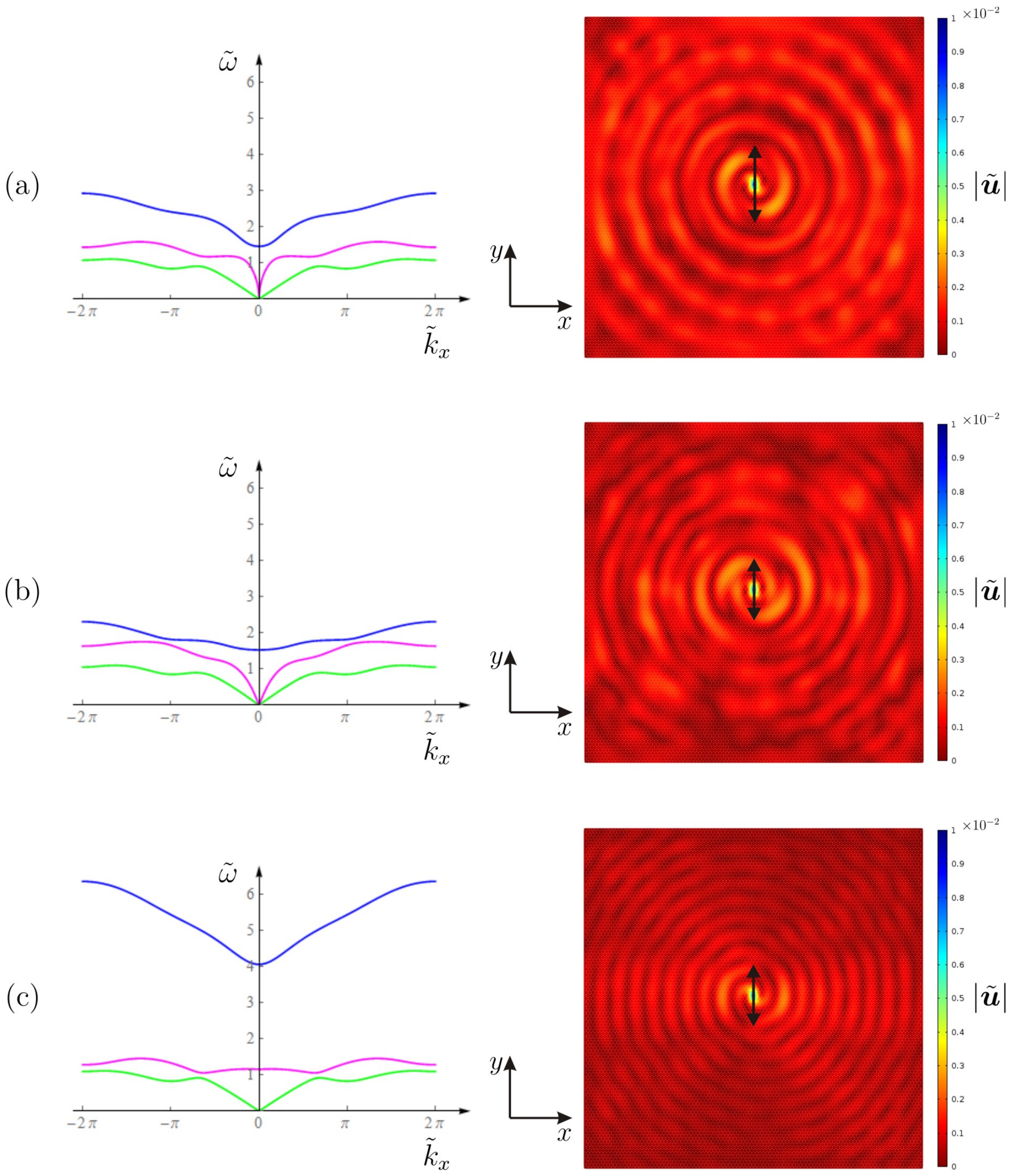

Figure S1: Cross-sections of the dispersion surfaces for $\tilde{k}_y = 0$ (left) and amplitude fields of the total normalised displacement, produced by a harmonic vertical displacement of low frequency $\tilde{f} = 0.05$ and amplitude 0.01 applied at the central node of the lattice (right), calculated for the following values of the spinner constants: (a) $\tilde{\alpha}_1 = 0.5$, $\tilde{\alpha}_2 = 1.5$, (b) $\tilde{\alpha}_1 = 0.1$, $\tilde{\alpha}_2 = 1.5$, (c) $\tilde{\alpha}_1 = 0.9$, $\tilde{\alpha}_2 = 1.5$. The finite element computations are performed on a lattice with an aspect ratio approximately equal to 1, having side length 100, surrounded by PML.

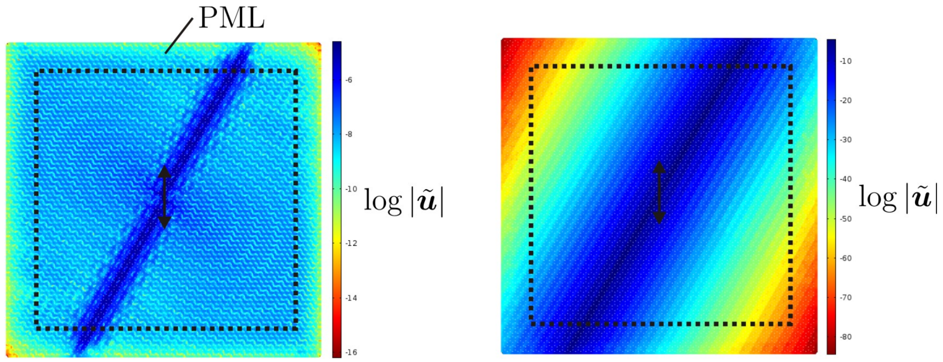

(a) (b)

Figure S2: Amplitude of the total normalised displacement in logarithmic scale for (a) $\tilde{\alpha}_1 = 0.9$, $\tilde{\alpha}_2 = -0.9$ and (b) $\tilde{\alpha}_1 = 0.8$, $\tilde{\alpha}_2 = -0.9$, when the lattice is subjected to a harmonic displacement of amplitude 0.01 (indicated by the arrow), at the Gaussian beam frequency. In the numerical simulations, the lattice is surrounded by PML.

Additional comments on visualisation of unidirectional waveforms

Figure S2a and S2b reproduce in logarithmic scale the amplitude fields of the total normalised displacement in the lattice, shown in Fig. 4b and 5b respectively. It is apparent that in the case when $\tilde{\alpha}_1 = 0.8$, $\tilde{\alpha}_2 = -0.9$ (Fig. S2b) the wave pattern is more localised, as already observed in the main text when describing Fig. 5b.

The strain energy in a lattice link averaged over a period T is defined in the time-harmonic regime as

$$E_s = \frac{1}{T}\int_0^T \int_V \frac{1}{2}E\left[\epsilon \sin\left(\frac{2\pi t}{T}\right)\right]^2 \mathrm{d}V\mathrm{d}t = \frac{1}{4}E\epsilon^2 Al, \tag{S6}$$

where E is the Young's modulus, A is the cross-sectional area, $V = Al$ is the volume and ϵ is the axial strain of each link. The normalised strain energy density, given by

$$\tilde{E}_s = \frac{1}{4}\epsilon^2, \tag{S7}$$

is plotted in Fig. S3a in linear scale and in Fig. S3b in logarithmic scale for the case $\tilde{\alpha}_1 = 0.9$, $\tilde{\alpha}_2 = -0.9$. In Fig. S3c $\tilde{E}_s$ is plotted in linear scale and in Fig. S3d we present $\log(\tilde{E}_s)$ for the case $\tilde{\alpha}_1 = 0.8$, $\tilde{\alpha}_2 = -0.9$.

The kinetic energy of a lattice particle averaged over a period is expressed in

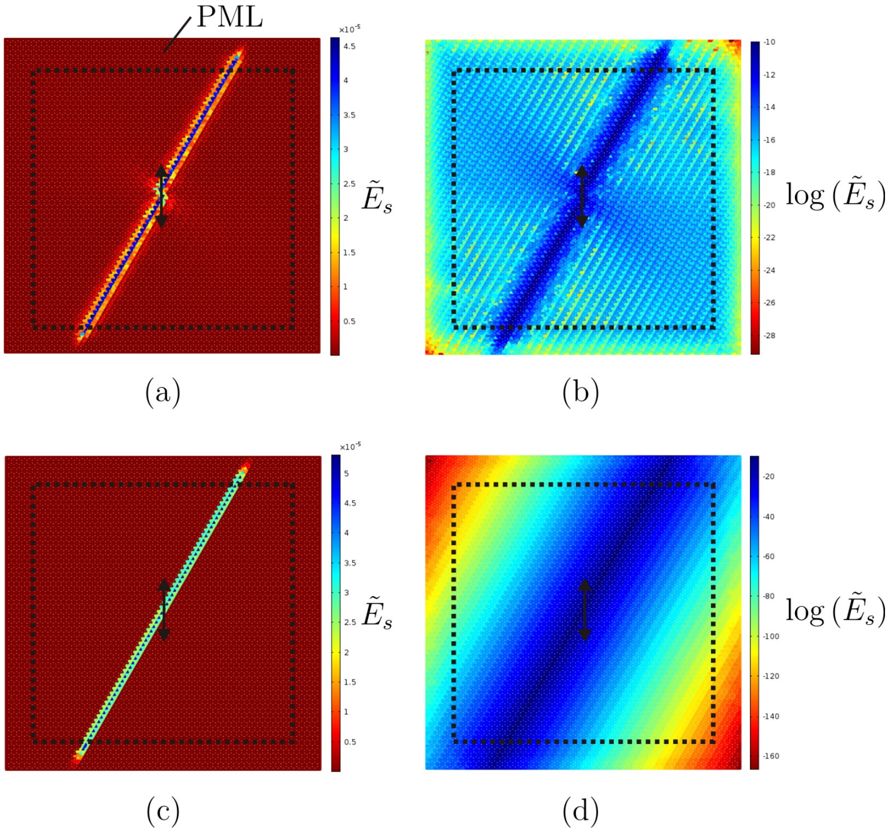

Figure S3: Amplitude of the normalised strain energy density in the lattice links for (a,b) $\tilde{\alpha}_1 = 0.9$, $\tilde{\alpha}_2 = -0.9$ and (c,d) $\tilde{\alpha}_1 = 0.8$, $\tilde{\alpha}_2 = -0.9$, in (a,c) linear scale and (b,d) logarithmic scale, due to a harmonic displacement of amplitude 0.01 at the Gaussian beam frequency.

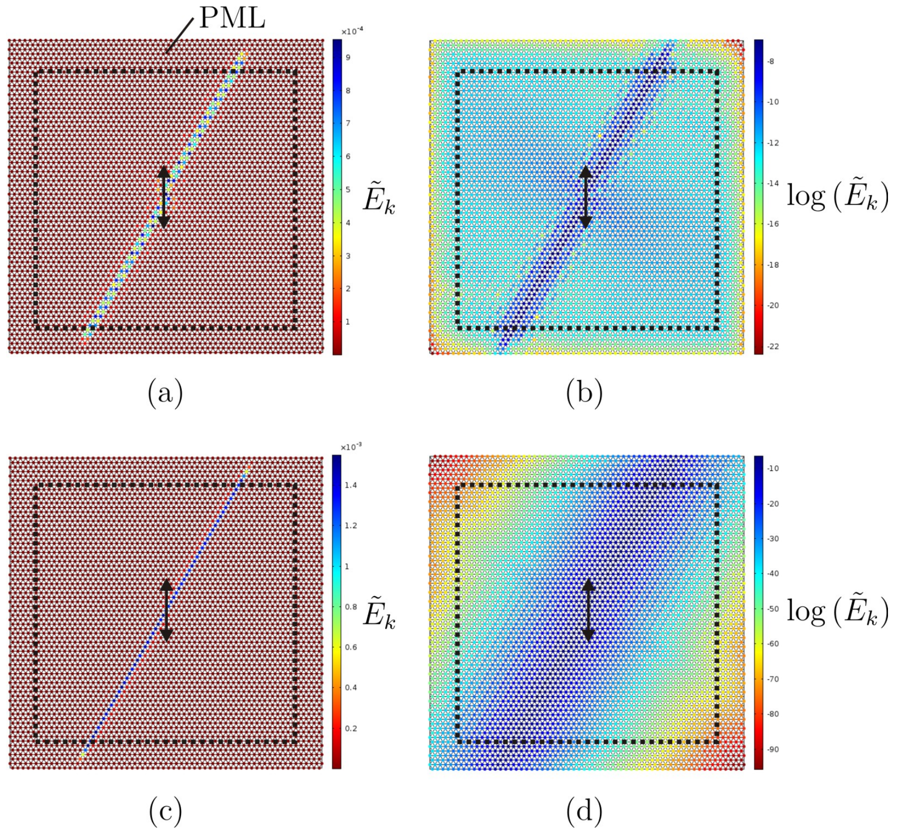

Figure S4: Amplitude of the normalised kinetic energy of the lattice particles for (a,b) $\tilde{\alpha}_1 = 0.9$, $\tilde{\alpha}_2 = -0.9$ and (c,d) $\tilde{\alpha}_1 = 0.8$, $\tilde{\alpha}_2 = -0.9$, in (a,c) linear scale and (b,d) logarithmic scale, due to a harmonic displacement of amplitude 0.01 at the Gaussian beam frequency.

the time-harmonic regime as

$$E_k = \frac{1}{T}\int_0^T \frac{1}{2}m\omega^2\left(u_x^2 + u_y^2\right)\sin^2\left(\frac{2\pi t}{T}\right)\mathrm{d}t = \frac{1}{4}m\omega^2\left(u_x^2 + u_y^2\right). \tag{S8}$$

The normalised kinetic energy

$$\tilde{E}_k = \frac{1}{4}\tilde{\omega}^2\left(\tilde{u}_x^2 + \tilde{u}_y^2\right) \tag{S9}$$

is shown in Fig. S4a in linear scale and in Fig. S4b in logarithmic scale for the case $\tilde{\alpha}_1 = 0.9$, $\tilde{\alpha}_2 = -0.9$. $\tilde{E}_k$ is plotted in linear scale in Fig. S4c and in logarithmic scale in Fig. S4d for the case $\tilde{\alpha}_1 = 0.8$, $\tilde{\alpha}_2 = -0.9$.

For each choice of the spinner constants, the normalised kinetic energy is larger than the normalised strain energy density, since the former depends on $\tilde{\omega}^2 \gg 1$. The amplitude fields of both strain and kinetic energy are more localised in the case $\tilde{\alpha}_1 = 0.8$, $\tilde{\alpha}_2 = -0.9$, as observed when discussing the displacement fields in Fig. 4 and 5.
